# Healthy obesity: time to give up the ghost?

**DOI:** 10.1080/03014460.2018.1444789

**Published:** 2018-04-11

**Authors:** William Johnson

**Affiliations:** School of Sport, Exercise and Health Sciences, Loughborough University, Leicestershire, UK

The notion that it is possible to be obese yet have no cardio-metabolic complications (e.g. dyslipidaemia and hyperinsulinemia) is attractive to those of us whose body mass index (BMI) has crossed the threshold of 30 kg/m^2^. However, since the first reports of so-called ‘metabolically healthy obesity’ in the 1980s, numerous studies have shown that such individuals (1) can be rare, depending on the population and diagnostic criteria, (2) transition to being unhealthy more frequently than their non-obese counterparts and (3) have increased risk of various non-communicable diseases (e.g. type 2 diabetes and chronic kidney disease) and higher mortality compared to healthy normal-weight individuals. Literature on the third point has been summarised in systematic reviews and meta-analyses, most of which have reached the same conclusion that healthy obesity is not benign. Despite the strength of this evidence, large-scale epidemiological studies are still frequently published on healthy obesity and disease/mortality risk in leading cardiovascular medicine journals. In September 2017, for example, the *Journal of the American College of Cardiology* (SJR 2016—11.488; impact factor 2017—19.896) published an analysis among 3.5 million adults, finding that healthy obese individuals had higher risk for incident cardiovascular disease events than their healthy normal weight peers (Caleyachetty et al., 2017). Naturally, the media have a field day in spinning these types of findings; the BBC news, in this instance, going with ‘Fat but fit is a big fat myth’, despite the study not looking at fitness (BBC, 2017).

What is less well-understood, or at least acknowledged, is why studies find increased disease/mortality risk in healthy obese individuals. As one might expect, individuals who are obese yet healthy are more likely to be younger, of European ancestry, lead a healthier lifestyle in terms of physical activity and diet, etc., have less central and visceral adiposity and be of higher socio-economic position than obese individuals who have already developed complications. The opposite is true (e.g. more likely to be older, non-European ancestry, lead an unhealthier lifestyle, etc.) when compared against a healthy normal-weight group. Thus, it probably comes as no surprise that levels of cardio-metabolic disease risk factors (e.g. systolic blood pressure and fasting glucose) are worse among healthy obese than healthy normal weight individuals, despite both groups having the same label of ‘healthy’. The vast majority of studies report these systematic differences in an initial descriptive statistics table, but then go on to conduct inferential analyses that ignore them. The problem arises from crudely dichotomising continuous variables to define weight and health status. For example, with a BMI cut-off of 30 kg/m^2^ and a blood pressure cut-off of 140/90 mmHg, both an obese person with blood pressure of 139/89 and a non-obese person with blood pressure of 110/70 would be classified as healthy. The fact that healthy obese individuals are actually less healthy on average than healthy normal weight individuals is intuitive and must, at least partly, explain why disease or mortality risk differs between the two groups. Documented associations of healthy obesity with disease or mortality risk might, therefore, be viewed as a self-fulfilling prophecy.

There is, of course, the argument that the concept of healthy obesity is clinically motivated, because it allows doctors to easily stratify the growing population of obese adults into those who most urgently require treatment and those who do not. Risks of disease and death are substantially lower among healthy obese individuals than unhealthy obese individuals, so targeting pharmacological and lifestyle interventions at the latter group would seem prudent given pressures on national health services. This must already happen to some extent in some healthcare settings. Nonetheless, an initial target of transitioning from an unhealthy to healthy cardio-metabolic profile, without necessarily losing a lot of body weight, has been described by Stefan et al. (2018) as the ‘low-hanging fruit’ in obesity treatment. As nicely surmised in their *Lancet Diabetes and Endocrinology* series paper, there is, however, limited evidence on which interventions would have this desired effect and there are various other hurdles to overcome before such a strategy could be implemented as standard care. One of these is agreeing on a universally accepted diagnosis for metabolic health; the conundrum being that existing binary definitions do not have great accuracy for predicting disease risk. Any intervention targeted solely at the unhealthy obese population would, therefore, miss many other ‘high risk’ individuals, not least those who are unhealthy but not obese.

The future of healthy obesity research might seem bleak from the picture I have painted, but it shouldn’t. The idea that different sub-groups of a population can have similar BMI values (and even similar levels of adiposity) but different cardio-metabolic disease risk factor profiles is both statistically credible, given rules of multivariate normal distributions, and biologically plausible (e.g. as an example of one mechanism, the same allele in a genetic variant near *ISR1* is related to both increased percentage body fat and decreased insulin resistance, dyslipidemia and risk of type 2 diabetes and coronary heart disease (Kilpelainen et al., 2011)). In my mind, however, the very construct of healthy obesity has led to a plethora of epidemiological research and debate on whether or not the phenomenon truly exists, instead of asking questions that accept, exploit and investigate heterogeneity among people with the same BMI. When viewed this way, there are many novel and important research questions that human biologists might see as being better aligned with their field. In particular, we know very little about the biological processes and mechanisms (e.g. growth and development patterns) and modifiable lifestyle factors (e.g. physical activity and diet), operating across the life course, that lead to some people developing a disease or dying while other people with the same BMI do not. Such research would help develop targeted prevention programmes, in line with various precision or stratified medicine initiatives, such as that of the UK Medical Research Council (MRC, 2010).

It is undeniable that obesity is bad for health, but there are clearly differences between individuals in the extent to which it is bad. While the concept of healthy obesity is crude and problematic and may best be laid to rest, there is great opportunity for human biological investigation of the levels, causes and consequences of heterogeneity in health among people with the same BMI.
